# Sialylation in the nervous system: Functions and mechanisms

**DOI:** 10.1016/j.jbc.2026.111288

**Published:** 2026-02-16

**Authors:** Kate Koles, Elena Repnikova, Boris Novikov, Vladislav Panin

**Affiliations:** 1 Department of Biochemistry and Biophysics, Texas A&M University, College Station, Texas, USA; 2 Department of Pathology and Laboratory Medicine, Children's Mercy Kansas City and University of Missouri-Kansas City School of Medicine, Kansas City, Missouri, USA

**Keywords:** sialic acid, sialylation, glycosylation, nervous system, neurotransmission, neurodegeneration, neurological disorders

## Abstract

Glycoprotein sialylation represents a critical posttranslational modification with diverse biological roles in animals. This review explores its multifaceted functions in the nervous system, with particular emphasis on neurophysiology, homeostasis, and associated neurological disorders. The sialylation pathway modulates key neural processes through effects on glycoprotein stability, localization, activity, and molecular interactions. Examples include its crucial role in the regulation of neuronal excitability by modulating the functions of voltage-gated ion channels. Recent studies have uncovered remarkably rapid, activity-dependent changes in synaptic sialylation, suggesting dynamic sialylation-mediated regulation of neural transmission and highlighting the importance of neuraminidases in these processes. Beyond synaptic function, sialylation mediates neuron–glia interactions through multiple mechanisms. It modulates immune functions regulated by siglecs and complement pathways while controlling microglial activation and neuroinflammation. The critical importance of proper sialylation is underscored by severe neurological manifestations associated with genetic defects in the sialylation pathway, including cognitive impairment, ataxia, and epilepsy. Furthermore, aberrant sialylation of glycoproteins and gangliosides has been implicated in neurodegenerative diseases (Alzheimer's and Parkinson's), brain cancers, and psychiatric disorders including schizophrenia and autism. Preclinical research has identified promising therapeutic strategies targeting sialylation. Studies demonstrate that polysialic acid administration reduces neurodegeneration, while siglec modulation alleviates age-related cognitive decline. Recent discoveries, including sialylated glycoRNA and insights from *Drosophila* models revealing unique sialylation-mediated glia-neuron crosstalk, have significantly expanded our understanding of this important regulatory system. These advances position sialylation as a promising therapeutic target for neurological disorders.

Glycosylation of major biologically important macromolecules, such as glycoproteins, glycolipids, and glycoRNA, is widespread in nature and commonly results in essential modifications of substrate functions (1, 2, 3). In metazoans, these glycosylated molecules are especially abundant in the extracellular milieu surrounding cell surfaces, collectively contributing to the glycocalyx, a prominent layer of glycoconjugates with complex yet specific effects on diverse cellular functions, influencing cell signaling, cell adhesion, pathogen–host interactions, and the biophysical properties of cells and tissues. Among different types of glycoconjugates, sialylated glycans are particularly notable due to the unique properties of sialic acid (Sia) residues. These residues comprise a family of negatively charged, bulky sugars typically attached to the termini of glycan chains *via* three distinct α linkages, α2-3, α2-6, and α2-8 (4, 5). Owing to these properties, Sia can critically affect glycoprotein functions through a range of mechanisms, including impact on glycoprotein stability and turnover, modulation of charge interactions, specific ligand recognition (such as binding to siglecs and other Sia-recognizing lectins), and functional masking by obstructing interactions with underlying glycan epitopes (6, 7). Sialylation is mediated by Golgi-localized sialyltransferases that use cytidine monophosphate sialic acid (CMP-Sia) as a sugar donor to modify glycan acceptors. CMP-Sia is generated by the evolutionarily conserved CMP-Sia synthetase (CSAS, also known as CMAS or CSS), which is a nuclear-localized enzyme in mammalian cells but is localized to the secretory compartments in *Drosophila* and other insects (8, 9, 10, 11). The mammalian sialylation pathway depends on the activity of a single CSAS generating CMP-Sia for subsequent utilization by 20 different sialyltransferases with distinct acceptor and linkage specificities which sialylate a multitude of substrates, including glycoproteins, glycolipids, and glycoRNA (2, 4, 12, 13). Many of these sialyltransferases have been characterized *in vitro* (14, 15, 16, 17, 18), however, how they function *in vivo* remains not well understood due to the complexity of mammalian glycosylation (7, 19, 20). Other enzymes of the sialylation pathway are also highly conserved in vertebrates and can contribute to the regulation of sialylation, including UDP-GlcNAc 2-epimerase/ManNAc kinase (GNE), a bifunctional enzyme that initiates the pathway, and N-acetylneuraminic acid phosphate synthase (NANS) (21, 22) that works downstream of GNE to convert N-acetylmannosamine-6-phosphate and phosphoenolpyruvate into N-acetylneuraminic acid-9-phosphate (Fig. 1). The catabolic branch of the pathway relies on the activity of neuraminidases (NEUs), also known as sialidases, the enzymes that trim sialic acid residues from glycoconjugates. Four neuraminidases, NEU1-4, with different localizations and expression patterns function in mammalian cells (23, 24). NEU-3 and NEU-4 show strong preference for gangliosides compared to glycoproteins, and all four enzymes differ in their relative preference for α2-3– *versus* α2-6–linked Sia (25, 26). Neuraminidases play vital yet incompletely understood biological functions by mediating remodeling of sialoglycoconjugates, and they have been implicated in a number of human diseases (26, 27). Recently, neuraminidases have been found to mediate key regulatory processes, affecting neural transmission and cell signaling in the nervous system (discussed below). For a thorough discussion of sialylation pathway genes and the genetic disorders associated with their defects, we refer readers to the accompanying review of this thematic series (Huang *et al. J Biol Chem, in press*).

**Figure 1:**
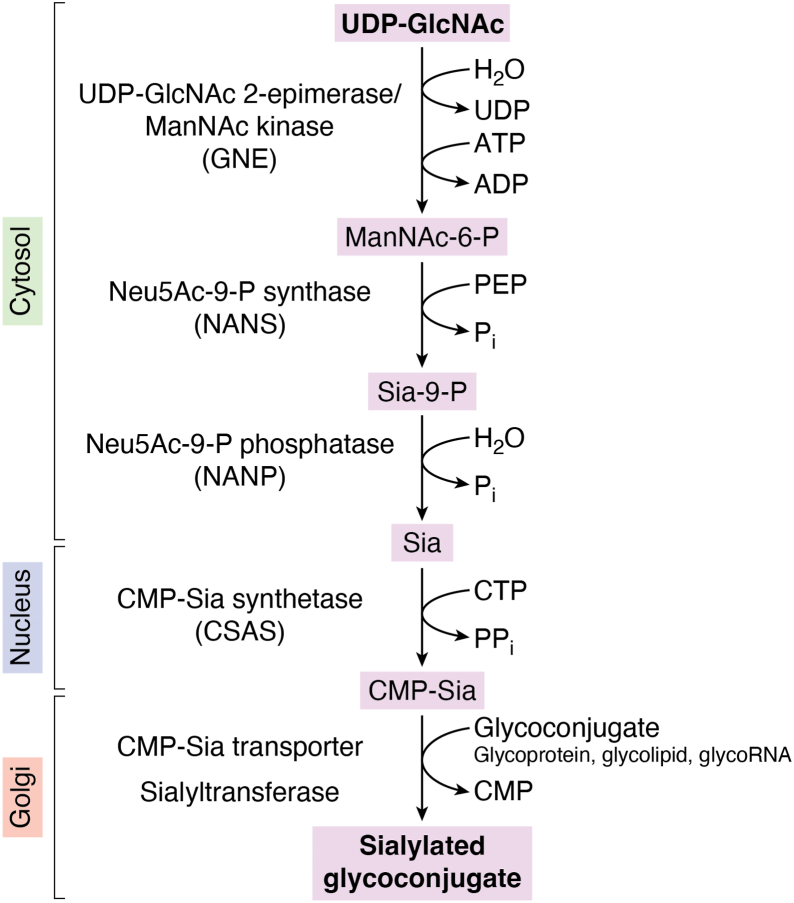
**Schematic of the sialylation pathway in vertebrates.** Subcellular localization of enzymatic steps is indicated on the *left*. Substrates and byproducts are shown on the *right*. UDP-GlcNAc, uridine diphosphate N-acetylglucosamine; ManNAc-6P, N-acetylmannosamine-6-phosphate; PEP, phosphoenolpyruvate; Neu5Ac-9P, N-acetylneuraminic acid-9-phosphate; Sia, sialic acid/N-acetylneuraminic acid; CMP-Sia, cytidine monophosphate sialic acid.

In humans, the level of sialic acid is highest in the brain due to the abundance of sialylated glycolipids (also known as gangliosides) on the neuronal cell membranes, carrying ∼75% of all sialic acids present in the brain (25, 28, 29). Gangliosides play vital roles during the maturation, maintenance, and pathology of the nervous system (see accompanying review in this thematic series (Mlinac-Jerkovic *et al. J Biol Chem, in press*) for detailed discussion). The repertoire of sialylated glycoproteins in the brain is also large, encompassing most cell adhesion molecules, ion channels, and other key players of the nervous system. Mutations in the sialylation pathway genes are associated with congenital disorders exhibiting pleiotropic phenotypes and complex clinical features, which usually include prominent neurological abnormalities, highlighting the critical role of sialylation in the nervous system. These disorders include severe conditions, such as intellectual developmental disorder (NANS defect), infantile epilepsy and intellectual disability (defects in ST3GAL3/5 sialyltransferases), developmental disability with ataxia (mutations in the CMP-Sia transporter), and hereditary inclusion body myopathy (mutations in GNE) (30, 31, 32, 33, 34). A recent study suggested that CMAS deficiency can also be associated with intellectual disability (35).

In our review, we will highlight the function of neural sialylation while focusing on the roles of sialylated glycoproteins in neural physiology, plasticity, homeostasis, and neuron–glia interactions. Although we will briefly address key developmental functions of sialylation and polysialylation, as well as the functions of proteins that specifically recognize sialylated structures (*e.g.*, siglecs), a more comprehensive discussion of these functions, as well as the thorough discussion of gangliosides and molecular mechanisms of polysialylation, can be found elsewhere and in the accompanying reviews of this thematic series dedicated to these topics ((36); Decloquement and Macauley, *J Biol Chem, in press*; Mlinac-Jerkovic *et al. J Biol Chem, in press*; Bhide and Colley, *J Biol Chem, in press*). Finally, we will discuss neurological conditions associated with defects in sialylation, as well as known and putative pathological mechanisms that underlie these disorders, along with potential therapeutic strategies.

## Developmental functions of glycoprotein sialylation

Genetic inactivation of Sia biosynthesis results in early embryonic lethality in mice, which highlights the essential role of sialylation in mammalian development (37). Crucial functions of sialylation in neural development have been uncovered by studying sialylation genes in various model systems, ranging from *Drosophila* to human cortical organoids (10, 31, 38, 39, 40). Important developmental roles of polysialic acid (polySia), a unique long polymer of sialic acid residues, concentrate on the regulation of neural cell adhesion molecule (NCAM) which is the main polySia carrier in the mammalian brain (41). PolySia-modified NCAM critically affects cell adhesion and has impact on many aspects of neural development, such as neuron migration, axon guidance and fasciculation, and synaptogenesis (41, 42, 43, 44) (*e.g.*, reviewed in (36)). Abundant evidence indicates that sialylated glycans not carrying polySia also play important roles in the development of the nervous system. Analyses of these roles in mammals are challenging because of pleiotropic effects of sialylation genes and complex regulation of glycosylation pathways, and thus only a few studies have pinpointed developmental functions of non-polySia-type sialylation in the mammalian nervous system. Beyond NCAM, many other cell adhesion molecules and guidance receptors in the developing nervous system carry more usual N- and O-linked sialylated glycans. Indirect observations indicate that this polySia-free sialylation is important for cell migration, axon pathfinding, and synaptogenesis. This evidence is largely based on the results obtained in different model systems (*e.g.*, cultured cells) that revealed effects of sialylation on cell adhesion and signaling molecules, such as integrins, epidermal growth factor receptor, and CD24 (45, 46, 47, 48). Thus, interactions of L1CAM with α2,3-linked sialylated glycans attached to the mucin-type cell adhesion glycoprotein CD24 were found to affect cerebellar and dorsal root ganglion neurite outgrowth in *ex vivo* primary cultures (48). Another example is the importance of sialylation for the function of endoglycan, a heavily sialylated mucin involved in axon guidance in the central nervous system (CNS) of chicken embryos (49). By analogy to polySia-NCAM–mediated cell repulsion, a sialylation-mediated mechanism of negative regulation of molecular interactions has also been proposed for endoglycan (49).

## Sialylation in neural physiology and signaling

Sialylation continues to function as a crucial regulator of the nervous system at postdevelopmental stages. In mature neurons and glia, sialic acids modulate electrical excitability, synaptic transmission, and cell–cell signaling on an ongoing basis. In this review, we will discuss major aspects of how glycoprotein sialylation influences neural function, including ion channel properties, synaptic plasticity, and interactions between neurons and glial cells.

### The impact of sialylation on ion channel function and neuronal excitability

**Figure 2:**
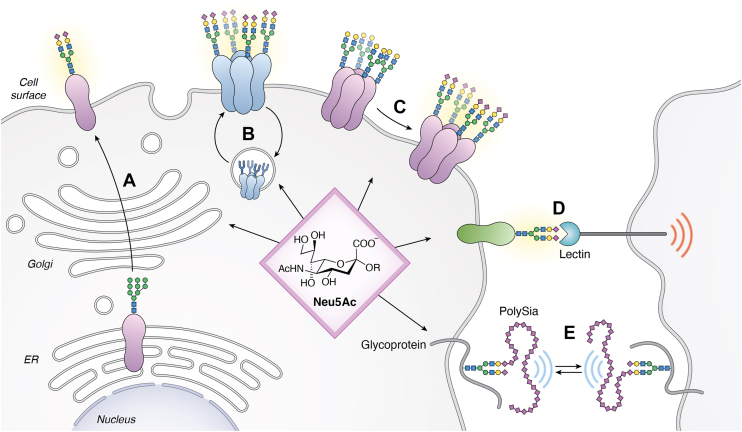
**Sialylation-mediated regulation of glycoprotein functions.** Sialylation can (*A*) promote subcellular trafficking of secretory pathway glycoproteins, (*B*) regulate turnover and recycling of secreted and membrane proteins between cell surface and endocytic compartments, (*C*) change biophysical properties and activity of glycoproteins *via* effects on protein conformation, steric, and charge interactions, (*D*) affect binding to lectins (such as siglecs) and modify cell adhesion and cell-signaling events, and (*E*) provide charge-mediated repulsion (usually involving polySia modification). A schematic of Neu5Ac, the most common type of sialic acid in human cells, is shown in the middle (OR, attachment to an underlying glycan). A common biantennary N-glycan structure (or a polySia-modified N-glycan in (*E*)) illustrates an example of glycoprotein sialylation, with individual sugar residues shown by symbolic representation (*blue square*, GlcNAc; *green circle*, mannose; *yellow circle*, galactose; *purplediamond*, sialic acid). See text for detailed discussion. Modified from (52).

Sialylated N-glycans have been found to affect the function of various voltage-gated ion channels, which are plasma membrane glycoproteins that serve as major determinants of cellular excitability. This mechanism underlies the effect of sialylation on membrane excitability, one of the most prominent functions of sialylation in the nervous system and heart (reviewed in (50, 51, 52)). Glycans can modulate functions of all major types of voltage-gated ion channels, including those transporting Na^+^, K^+^, and Ca^2+^, in a cell type- and developmental stage-specific manner (*e.g.*, (53, 54, 55, 56, 57, 58, 59, 60)). The channel functions can also be modulated by glycosylation of channel-interacting glycoproteins. For example, glycans on channel auxiliary subunits can promote cell surface localization or modify channel biophysical properties and affect channel gating (57, 61, 62, 63). The presence of Sia can play a major part in these glycan-mediated effects on channel functions (*e.g.*, reviewed in (50, 51)). Their negative charge and terminal location on carbohydrate chains promote interactions with ions and other molecules at the cell surface and within the secretory and recycling pathway compartments, thus affecting channel functions, trafficking, and stability (Fig. 2).

Vertebrate voltage-gated Na^+^ channels (Na_V_s) are among the best studied channel proteins. They are heavily glycosylated, having up to 30% of the molecular mass contributed by glycans, including sialic acids that can represent nearly 50% of that contribution (64, 65, 66, 67, 68). Na_V_ is composed of an ion selective pore-forming alpha subunit bound by two auxiliary beta subunits. Numerous Na_V_ variants composed of different subunit isoforms are expressed in various cell types. Electrophysiological assays have revealed a prominent effect of sialylated glycans on Na_V_ function, which varies for different channel isoforms (55, 56, 69). These results have been generally explained by the electrostatic effect of Sia residues located near the channel pore, consistent with the observation that N- and O-linked sialylation can have additive effects on channel gating (70). The impact of polySia on channel function is also well documented. Vertebrate Na_V_ can bear more than 100 Sia residues, mainly represented by polySia (64, 65, 71). The role of polySia in Na_V_ regulation was demonstrated in mouse cardiomyocytes with genetically inactivated ST8Sia II polysialyltransferase, a glycosyltransferase involved in polySia biosynthesis. The ST8Sia II mutation resulted in cell excitability defects, including abnormal Na_V_ gating and defects in action potentials (72). However, polySia may not uniformly impact Na_V_ functions in all cellular contexts. For example, removing polySia with Endo-N sialidase in rat hippocampal slice cultures did not affect action potentials and evoked synaptic transmission (73). Importantly, sialylation not involving polySia production can also play a prominent role in channel modulation, while polySia and polySia-free sialylation may have distinct effects on Na_V_ function. Thus, mutations that differentially impact sialylation and polysialylation were found to have distinct effects on the gating properties of Na_V_1.4 expressed in Chinese hamster ovary cells (74, 75). Furthermore, the impact of Sia on voltage-gated channels can go beyond electrostatic effects, as metabolic incorporation of Sia with N-pentanoyl or N-propanoyl structures substituted for N-acetyl groups affected the conductance of the Kv3.1 voltage-gated K+ channels, suggesting that sialylation can modulate channels by specific molecular interactions and steric effects (76). Recent cryo-EM structural information combined with molecular modeling and molecular dynamics simulations indicated that sialic acid residues of N-glycans attached to extracellular turret loops of Na_V_ channels may interact with voltage-sensing domains, which potentially underlies the effect of sialylation on channel biophysical properties (77). The structural analysis suggested that sialylated glycans can also affect interactions with the Ig domains of β subunits, thus promoting channel clustering and thereby having important consequences for cell electrophysiology (77).

Voltage-gated potassium channels (K_v_) are involved in restoring the membrane potential to its resting levels after action potential propagation, which is essential for neuronal signaling. Similar to sodium channels, K_v_ channels are decorated with N- and O-glycans. Analyses of primary cultured hippocampal neurons revealed that nonglycosylated K_v_1.2 channels were degraded faster upon internalization (78). Notably, this effect of glycans largely depends on sialylation, as Sia on a conserved N-glycan site in the first extracellular loop of the channel α subunit was essential for preventing channel degradation and promoting channel stability on the cell membrane (78). Intriguingly, recent studies have suggested that Sia can be added extracellularly, challenging the long-held assumption that sialylation occurs exclusively through cell-autonomous mechanisms within the Golgi of cells that produce both the sialyltransferase and its glycoprotein substrates (79, 80, 81). This so-called “extrinsic sialylation,” mediated by secreted or transacting membrane-bound sialyltransferases utilizing extracellular CMP-Sia, potentially provides a mechanism for rapid glycan-mediated regulation of neural signaling. Thus, a novel approach, *in situ* glycan editing, has uncovered that α2-3 sialylation of the human Ether-à-go-go-Related Gene (hERG) Kv channel could be added *in situ* by a soluble sialyltransferase. This modification resulted in altered channel gating, specifically requiring a higher depolarization voltage to activate hERG (82). Cell-nonautonomous remodeling of cell surface sialylation by secreted sialyltransferase is thought to play important roles in the immune system (83). Although this mechanism has not yet been demonstrated in the nervous system, it could potentially facilitate dynamic glycan remodeling on neuronal membranes, including in distal neurite regions and at synapses, thereby enabling rapid modulation of neural transmission (84).

It is worth noting that some studies demonstrating the importance of glycosylation for channel functions in the nervous system did not distinguish the effects of glycans from those of sialylation *per se*. For instance, glycan modifications were shown to be attached to the outer pore loops and be important modulators of TRPM8, a member of the transient receptor potential ion channel family playing essential roles in sensory physiology of temperature responses (85, 86). Glycans were also found essential for the agonist-mediated regulation of TRPV1 (TRP vanilloid type 1), a nonspecific cation channel that functions as a sensor of painful stimuli (87). It is tempting to speculate that Sia plays key roles in these glycan functions; however, further detailed structure-function analysis of glycans and the specific role of sialylation is required to test this hypothesis.

### Sialylation in receptor trafficking, localization, and stability

Similar to the effects on ion channels, glycosylation—and particularly sialylation—can modulate different receptor proteins, affecting their interactions with ligands and other binding partners, their association with the extracellular matrix, and their regulation by factors that control trafficking, subcellular localization, and activity. These effects have been demonstrated for several glycoproteins with important functions in the nervous system. For instance, sialylation mediated by ST8Sia3, a sialyltransferase that modifies glycoproteins with short α2-8–linked repeats of Sia residues (di- and tri-sialylated structures), has been shown to affect the spatial distribution of its substrates (88). Several G-protein–coupled receptors enriched in the striatum, including adenosine receptor A_2A_ and dopamine receptor D_2_, are substrates of ST8Sia3. Lack of ST8Sia3-mediated sialylation increases the distribution of A_2A_ and D_2_ receptors into lipid rafts, specialized regions of plasma membrane where diverse proteins are selectively concentrated and participate in signaling. Given that A_2A_ and D_2_ form heteromers with mutually antagonistic effects on each other (89), these findings suggest that sialylation can regulate these receptors by inducing their subcellular redistribution, decreasing their interactions, and thereby promoting their functions. The A_2A_–D_2_ pathway affects locomotor activity and is an important drug target for neurological disorders, such as Parkinson's disease and schizophrenia, highlighting the significance of this regulation (89). Similar mechanisms potentially regulate other sialylated glycoproteins by changing their cell membrane distribution and affecting their molecular interactions. Although beyond the scope of this review, it is important to note that gangliosides also play a crucial role in this context, exerting prominent effects on membrane microdomain formation and electrostatic potential at the synapse ((28, 29) and the accompanying review (Mlinac-Jerkovic *et al. J Biol Chem, in press*).

### The emerging roles of dynamic sialylation in network activity, synaptic transmission, and plasticity

While neuronal network activity and synaptic plasticity are thought to be mediated primarily by interactions of neurons, recent studies have also highlighted the essential contribution of glia in these processes and revealed the involvement of the sialylation pathway in their regulation. We will discuss the role of sialylation in neurons and glia at different functional levels, from neuronal networks to synapses, and highlight the involvement of neuraminidases in functional remodeling of neural sialylation, while focusing on new developments and ideas in this research area.

The importance of proper membrane sialylation for proper neuronal network activity was demonstrated earlier using electrophysiological recordings from rat hippocampal slices and treatment with a neuraminidase that nonspecifically cleaves terminal sialic acid. These experiments showed that the reduction in cell surface sialylation modified the kinetic parameters of Na_v_ channels and action potential threshold of hippocampal CA3 pyramidal cells, which changed the network seizure threshold and had prominent anticonvulsive effects (90). More recent experiments using cultured hippocampal cells confirmed these conclusions, while also revealing that different sialic acid linkages on distinct molecules can exert dissimilar effects on neural network functioning (91). The targeted removal of α2-3–linked sialic acids using a linkage-specific neuraminidase unexpectedly resulted in increased network firing associated with a decrease in action potential threshold (91). The ablation of α2-6 sialylation resulted in the loss of functional connectivity of neurons, while removal of α2-3–linked Sia only minimally perturbed it. Given that in the nervous system α2-3–linked Sia is most abundant on gangliosides, these linkage-specific effects imply distinct contributions of sialylation of glycoproteins and glycolipids to neural network functions.

Another line of evidence for the role of sialylation in neuronal networks was revealed in a study using human neurons derived from induced pluripotent stem cells (40). Sialylation was inhibited by a fluorinated synthetic analog of sialic acid (SiaFEtoc), which greatly reduced the amount of polySia without affecting cell differentiation and dendrite outgrowth. At the same time, this caused a reduction in functional synapses, induced both the pre- and post-synaptic defects, and significantly influenced network parameters, resulting in lower firing rate, increased random activity, and shorter network bursts. These phenotypes suggested causative defects in NCAM polysialylation (41) and the function of neurotransmitter receptors, including NMDA and AMPA receptors. These receptors are heavily glycosylated, including modifications with sialylated N-glycans. The glycosylation of NMDA and AMPA receptors is known to be important for their function, affecting trafficking, cell surface localization, the formation of functional molecular complexes, and modifying biophysical properties (92, 93, 94, 95, 96). Collectively, these findings suggested that sialylated glycans play a critical role in modulating the function of these receptors and supported the hypothesis that aberrant sialylation of neurotransmitter receptors substantially contributes to disruption in neural network activity (40).

Endogenous neuraminidases may be able to shape the properties of neural networks by decreasing neuronal sialylation, while also linking immune responses to neural functions. Exposure to bacterial lipopolysaccharide was found to induce microglia-mediated production of extracellular vesicles with neuraminidase 3 (Neu3) (97). The fusion of these vesicles with neuronal membranes resulted in cell surface desialylation and disruption of neuronal network connectivity. This Neu3-mediated remodeling of neuronal glycocalyx leading to aberrant network-level neuronal activity may have important implications in neuroinflammatory disorders, such as Parkinson's and Alzheimer's diseases. These findings, as well as data from other studies, underscore the important role of sialylation in the formation, maintenance, and plasticity of neuronal networks.

While effects on neural network properties are traditionally considered on a timescale of hours or days, there is growing evidence that changes in sialylation can induce effects on timescales as short as a few seconds. A recent study revealed that neuraminidase activity in the rat CA3 stratum lucidum was increased in response to long-term potentiation–inducing high-frequency stimulation within several seconds (98). The level of free sialic acid in the hippocampal extracellular space was elevated during hippocampus-dependent memory formation, suggesting that neural activity–dependent desialylation was involved in hippocampal memory processing and synaptic plasticity (98). Remarkably, inhibition of the spontaneous firing rate of neurons under basal, nonstimulated conditions resulted in decreased desialylation, suggesting that sialidase activity can be upregulated even by spontaneous spikes. Astrocytes also contributed to this process, however, the underlying mechanism remains to be elucidated (98).

Another important insight into the surprisingly fast and specific nature of changes in synaptic sialylation was achieved by quantitative glycoproteomic analysis of N-linked sialylated glycosites (also known as “sialiomics”) of glycoproteins isolated from rat synaptosomes subjected to a brief (5-second duration) potassium-induced depolarization. The results uncovered striking changes in the sialylation status of more than 400 N-glycosites on numerous synaptic glycoproteins, including neurotransmitter receptors (GABA and glutamate receptors), ion channels and transporters (such as voltage-dependent calcium channels, sodium/potassium transporting ATPases, potassium voltage-gated channels, sodium/potassium/calcium exchangers), synaptic vesicle proteins (*e.g.*, synaptophysin, synaptoporin), and cell adhesion molecules (*e.g.*, contactins, neural cell adhesion molecules, neuroligin) (99). Desialylation was found to be more prevalent than sialylation, and several neuraminidases and sialyltransferases capable of mediating these modifications were identified within the isolated synaptosome fractions. These data are consistent with the above-mentioned activity-dependent fast changes in desialylation in the rat hippocampus, reinforcing the idea that (de)sialylation can be a very rapid posttranslational modification, in addition to its canonical functioning on a much longer time scale. These results also raised questions about possible mechanisms of the activation of (de)sialylation enzymes (*i.e.*, sialyltransferases and neuraminidases) on such a short time scale. An example of such a mechanism was reported for Neu1/3 activation by local milieu pH changes induced by NHE1 Na+/H+ antiporter in epithelial cells in response to epidermal growth factor signaling (100). The scenario of pH-induced activation of neuraminidases in neural transmission is consistent with the narrow morphology of the synaptic cleft that restricts proton diffusion, creating local environment conducive to fast changes in proton concentrations. Voltage-gated proton channels and Na^+^/H^+^ exchangers expressed by neurons and glia could generate the local pH changes that lead to the activation of neuraminidases. Although this mechanism is supported by activity-dependent transient acidification at synapses detected in primary cortical neurons (101), alternative pathways of rapid neuraminidase activation, akin to the process of Neu1 activation by molecular interactions of neurotrophin with Trk tyrosine kinase receptors (102), remain plausible. Comprehensive studies are necessary to elucidate the precise molecular mechanisms underlying activity-dependent modulation of synaptic glycoprotein sialylation. Importantly, changes in the sialylation status of gangliosides, such as those induced by neuraminidases, have also been implicated in the critical modulation of neural activity, synaptic function, and axon regeneration (97, 98, 103, 104, 105). However, these mechanisms are outside the scope of this review, and we refer readers to the accompanying review on ganglioside functions for detailed discussion (Mlinac-Jerkovic *et al. J Biol Chem, in press*).

Taken together, recent findings highlight that regulated neural sialylation plays an essential role in modulating neural circuit activity and plasticity on a wide range of timescales. By dynamically modifying the “glyco-code” of receptors and channels during activity, neurons can rapidly adjust synaptic strength and network functioning in an activity-dependent manner. At the same time, long-term changes in the sialylation of glycoproteins and gangliosides may establish distinct functional states of neuronal networks that can affect neural functions over long periods of time. Overall, these results support an emerging paradigm that the nervous system is extensively regulated by dynamic and widespread changes in sialylation.

## Sialylation in neuron–glia interactions and immune regulation

Immune responses in the nervous system depend significantly on a delicate balance between sialylation and sialic acid–recognizing molecules that translate this recognition into immune modulation. Siglecs, short for “sialic acid-binding immunoglobulin-like lectins”, and the complement factor H (CFH), a key player of the innate immune system, are among the most important sialic acid–recognizing molecules in immune responses (106). Through dynamic interactions with the sialiome, siglecs and CFH modulate cell signaling and affect numerous aspects of neuroinflammation, autoimmunity, neurodegeneration, while also affecting homeostasis and aging.

### Siglecs and PolySia

Siglecs make up a family of transmembrane proteins characterized by the presence of a V-set Ig domain at the N terminus responsible for sialic acid recognition. They are primarily expressed on the surface of innate immune cells, including microglia, the resident cells of the brain that are critical for neural development, repair, and homeostatic regulation of neuronal networks (107, 108). Within their intracellular tails, “inhibitory” siglecs have immunoreceptor tyrosine-based inhibitory motifs that recruit phosphatases when stimulated, thus affecting cell signaling. When engaged by sialic acids on the surface of healthy neurons or microglia, immunoreceptor tyrosine-based inhibitory motif–containing siglecs expressed on immune cells transmit an inhibitory signal maintaining the cell in a quiescent state. Reduction in cell surface sialic acids, for example, due to injury, infection, aging, or neurodegenerative conditions, can downregulate the activity of inhibitory siglecs, thus contributing to microglial activation. However, another class of siglecs, termed “activating,” can trigger the opposite effect *via* an activating signaling cascade by recruitment of DAP12, an adaptor protein containing an immunoreceptor tyrosine-based activation motif that signals through tyrosine kinases to induce cell activation. Although the functions of siglecs have been extensively studied and generally characterized, their molecular and cellular mechanisms are complex and remain not fully understood. For more detailed discussion of siglecs' structure-function relationships, we refer to the accompanying review in this thematic series (Decloquement and Macauley, *J Biol Chem, in press*).

Mouse CD22, a conserved member of the siglec family, was identified in a genetic screen as a negative regulator of phagocytosis in aged microglia. The antiphagocytic effect of CD22 is mediated by interactions with α2-6–linked sialylated ligands, and inhibition of CD22 can restore microglial homeostatic functions in the aging brain, promoting the clearance of myelin debris and removal of pathogenic α-synuclein fibrils and beta-amyloid (Aβ) oligomers (109). The role of microglia in neurological homeostasis, maintenance, and regeneration of myelination is conserved in the human nervous system (110), however, whether CD22 has similar functions in the aging human brain remains to be elucidated. Further studies in this area are expected to have important implications for the treatment of neurodegenerative disorders and provide insights into the mechanisms of brain aging.

PolySia-mediated inhibition of murine microglia was found to depend on interactions with Siglec-E, while neuropilin-2 or E-selectin ligand-1 have been identified as relevant polySia carriers that are released by shedding in response to inflammatory activation (111). More recently, using cerebellar organotypic slice cultures as a model for myelin repair, it was demonstrated that exogenously applied soluble polySia could inhibit inflammatory microglia and significantly improve remyelination in a Siglec-E dependent manner (112). Human-specific inhibitory Siglec-11 and its paired activating receptor Siglec-16 also bind polySia and are thought to be engaged in functionally analogous neuroinflammatory responses in the human nervous system (111, 113, 114). However, most people are homozygous for a nonfunctional allele of *Siglec16* and the biological significance of this gene remains not well understood (115). The importance of the polySia–Siglec-11/16 axis from biomedical perspectives was highlighted by a study demonstrating that soluble polySia can ameliorate laser-induced damage in mice retina with the transgenic expression of human Siglec-11, suggesting a promising therapeutic strategy for age-related macular degeneration (116). Furthermore, activation of tumor-associated microglia and macrophages in polySia-positive glioblastoma tumors were found to correlate with tumor elimination and increased survival of patients with functional Siglec-16 (117), which opens a prospective avenue for developing improved treatments of polySia-positive tumors in the nervous system (118).

To further illustrate the intricacies of Siglec and polySia-mediated effects on the nervous system, it is worth pointing out that human microglia express a unique isoform of Siglec-11 that has a higher affinity for polySia than the macrophage-specific variants. This isoform can be secreted either cleaved or in full-length, released *via* exosomes, and has the potential to function at a distance (119). In addition, polySia expression in response to inflammatory signaling can change rapidly, within 10 to 30 min, while being degraded by Neu1, a secreted endogenous neuraminidase released on exosomes (120). This degradation leads to the release of polySia-bound brain-derived neurotrophic factor, which may underlie a mechanism of supplying trophic molecules at sites of injury. However, polySia degradation can also have a negative effect on recovery from brain injury, as endogenous neuraminidase activity was found to interfere with the migration of new neurons to the site of injury by reducing polySia and increasing cell adhesion which hampers neuronal regeneration (121). All these examples highlight mechanisms that introduce additional layers of sialylation-mediated regulation in healthy and disease states, highlighting their significance for potential therapeutic interventions in conditions associated with brain injury and inflammation.

### Interactions between complement and sialic acid in the nervous system

While the functions of the complement system and siglecs are aligned and deeply intertwined, for clarity, we discuss sialic acid–mediated complement interactions in this separate section.

The complement system is a crucial part of the innate immune system consisting of over 30 proteins that work together to eliminate pathogens, clear damaged cells, and enhance immune responses, while also playing important roles beyond canonical innate immunity (122). CFH is one of the most important regulatory proteins in this system, helping prevent excessive complement activation and protect healthy host tissues from damage. Factor H works through several mechanisms, including sialic acid recognition (123).

Complement receptor 3 (CR3 or CD11b/CD18) on microglial surfaces mediates immunoreceptor tyrosine-based activation motif signaling *via* DAP12, eventually leading to phagocytosis. The complement cascade is initiated by the microglial release of C1q and C3, opsonins that tag cells or debris for phagocytosis. Such tagged targets can undergo phagocytosis induced by the CR3 receptor that detects iC3b-opsonized targets. Under noninflammatory conditions, this microglial activation is counterbalanced by the effect of CFH that binds to α2-3–linked sialic acids (among other ligands) and promotes the degradation of C3 convertase and C3b (106, 123). However, when microglial cells are activated, for example, by lipopolysaccharide or Aβ (fibrillar amyloid beta) exposure, they release neuraminidase that desialylates cell surfaces, which further stimulates phagocytosis of neurons (124). This indicates that secreted neuraminidase and CR3 can be potential therapeutic targets to prevent inflammatory loss of neurons. The complement-mediated homeostatic loss of synapses and neurons induced by hyposialylation and microglia activation was demonstrated in mouse models with genetic downregulation of sialylation, suggesting that related pathomechanisms can operate in neurodegeneration and aging in humans (125, 126).

Another important connection between the innate immunity pathway and the nervous system is mediated by TREM2 (triggering receptor expressed on myeloid cells 2), a multifunctional innate immune receptor of immunoglobulin family. TREM2 was found to have complex glycosylation required for the receptor stability, trafficking, and signal transduction (127). TREM2 is involved in microglia activation and synapse elimination during brain development, while TREM2 abnormalities are associated with several neurological conditions, such as dementia, Nasu-Hakola disease, autism, and an increased risk of Alzheimer's disease. TREM2 deficiency in mice causes abnormal brain connectivity due to defect in developmental supernumerary synapse elimination, resulting in autism-like behavior (128). Mouse TREM2 is downregulated *via* sialylation that leads to accumulation in microglia, including lysosomes and at the plasma membrane, and prevents activation of SYK (spleen tyrosine kinase) and downstream signaling, which eventually precludes TREM2-dependent phagocytosis (129). Neu1-mediated desialylation of TREM2 regulates its proteolytic processing, modifies interaction with DAP12, and promotes downstream processes, such as Akt signaling, NF-κB inhibition, NFAT1 activation, and phagocytosis (129). This mechanism suggests that NEU1 modulation can be a therapeutic strategy to control microglia-mediated neuroinflammation in a number of diseases, including Alzheimer's and sialidosis (129).

### GlycoRNA and siglecs

A recently discovered new member of the glycoconjugate family, glycoRNA, is further deepening our understanding of the structural complexity and functional variation afforded by glycosylation, in particular by sialylation. Using Ac_4_ManNAz (N-azidoacetylmannosamine) for metabolic labeling of sialylated glycoconjugates, Flynn et al. identified short RNAs modified with sialylated N-glycans (2). These glycoRNAs are membrane associated and trafficked to the cell surface. Interestingly, Siglec-11 and Siglec-14 can bind to cell surface glycoRNA, suggesting new players in immune regulation. Y-RNA, a type of short RNAs enriched in the brain (130), was found among the most abundant sialylated glycoRNAs (2), while Siglec-11 is highly expressed in microglia, which further supports the scenario that their interactions can potentially modulate immune processes and homeostasis in the brain, affecting aging and neurological conditions, such as neurodegeneration. Recent studies indicated important roles of glycoRNA in organizing cell-surface domains involved in cell communication and mediating innate immune modulation in cell culture and mouse models (3, 131), suggesting that glycoRNA potentially plays similar roles in the nervous system. Future studies in this new field are expected to uncover novel important roles of sialylation in glycoRNA-controlled processes in the nervous system.

### Homeostatic plasticity, stress response, and aging

The role of sialylation in homeostatic plasticity, neural stress responses, and aging is significantly intertwined with the mechanisms regulating neuroinflammation and microglia function. As discussed above, sialylation shields neurons from the destructive effects of microglia and complement by engaging inhibitory siglec receptors on microglia and blocking binding of complement components, thus protecting synapses from abnormal trimming (125, 126). PolySia is also thought to play a key role in homeostatic regulation and stress responses. A brief acute stress in rats can trigger transient and long-lasting changes in brain polySia in a region-specific manner and without changes in NCAM levels (132). In agreement with the involvement of microglia-secreted neuraminidase in remodeling cell surface sialylation (120), the stress-associated polySia changes in the olfactory bulb have been suggested to be induced by microglia-mediated neuraminidase production. Interestingly, the stress-induced polySia remodeling in the prefrontal cortex was mediated by neuraminidase(s) expressed by astrocytes, suggesting that glia play a major role in controlling neuronal sialylation in a region-specific manner, mediating homeostatic regulation of neural transmission and responses to stresses (132).

Decline in polySia levels was found in aged mouse brain, while no decrease in overall Sia was detected, suggesting that polySia plays a particularly important role in aging (133, 134). Accelerated aging and related decline in brain functions are also common features of many neural pathologies, such as Alzheimer's disease (AD). Remarkably, intranasal administration of short polySia fragments (12 Sia residues, NANA12) was able to rescue neurological phenotypes in mouse AD models, including decline in cognitive task performance and an imbalance in synaptic/extra-synaptic NMDAR (N-methyl-D-aspartate receptor) signaling (135). In a recent follow-up study, the administration of NANA12 was found to improve cognitive functions in aged mice, which was associated with normalized size of microglial lysosomes and reduced phagocytosis of synaptic proteins (reported in a preprint (136)).

**Figure 3:**
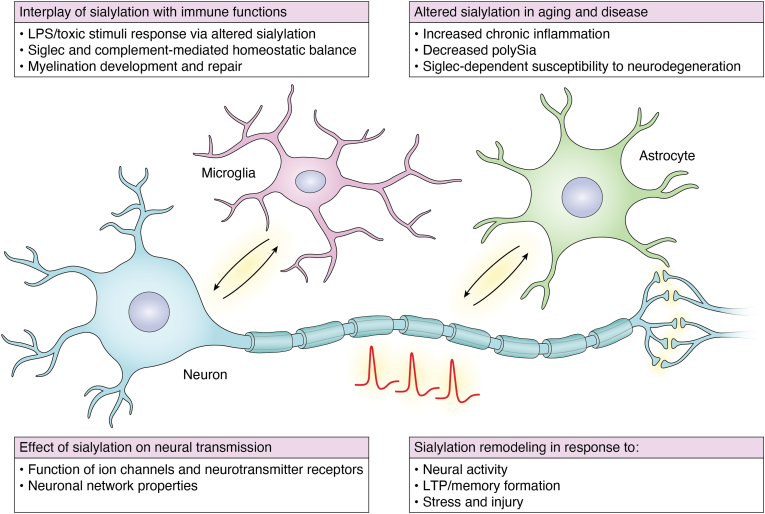
**Mechanisms associated with neural sialylation.** The mechanisms include interplay with immune system functions, effects on neural physiology and homeostasis, and involvement in aging and disease. Dynamic remodeling of sialylation under different conditions can affect most of these functions. Background schematic: sialylation can affect several cellular functions and interactions in the nervous system, including neuron–glia interactions (involving microglia and astrocytes) and neuronal signaling through modulation of cellular excitability and synaptic transmission. See text for detailed discussion.

In summary, an increasing amount of data indicates that sialylation, particularly polySia, is critical for enforcing a homeostatic balance in neural networks, facilitating synaptic remodeling and circuit scaling under stress, protecting synapses from degeneration, and supporting neural functions during aging (Fig. 3). Microglia play pivotal roles in these processes. However, we are only beginning to understand the extent to which sialylation is integrated into various cellular and molecular mechanisms that underpin microglial functions and the role of the immune system in brain development and homeostasis.

## Evolutionary perspectives on sialylation: Insights from the *Drosophila* model

**Figure 4:**
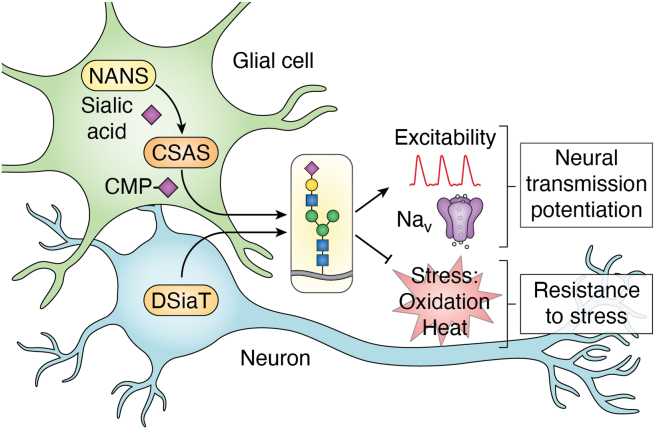
**Sialylation-mediated glia-neuron coupling in the Drosophila nervous system.** Sialic acid and CMP-Sia are produced by NANS and CSAS, respectively, in glial cells, while the sialyltransferase (DSiaT) is expressed in neurons. This bipartite arrangement of the sialylation pathway enables glia-mediated control of neural sialylation, which regulates neuronal excitability, Na_V_ channel (Para) maintenance, and stress responses. Modified from (144).

New insights into neural sialylation have been obtained by studies in the *Drosophila* model. *Drosophila* has a simplified sialylation pathway mediated by the genes with clear orthologous relationship to mammalian counterparts, including NANS, CSAS, and sialyltransferase, which makes fruit flies a useful model to investigate the evolutionarily conserved functions of sialylation (9, 10, 39, 137, 138, 139). *Drosophila* has a single sialyltransferase, DSiaT, that is closely related to mammalian ST6Gal sialyltransferases modifying glycoconjugates with α2-6–linked sialic acid (137). The activity of sialylation genes is restricted in *Drosophila* to the nervous system (10, 39, 137). Also, the *Drosophila* sialylation pathway lacks the ability to produce polySia or gangliosides, making it a suitable model for investigating the role of polySia-free sialylated glycans in the nervous system (140). Genetic and biochemical approaches demonstrated that the *Drosophila* repertoire of sialylated structures is largely represented by hybrid-type N-linked glycans (141, 142), while functional *in vivo* analyses uncovered the role of sialylation in the regulation of neural excitability and synaptogenesis, including involvement in the development of synaptic connections at neuromuscular junctions (10, 39, 143). Genetic inactivation of sialylation genes results in significantly shortened lifespan and prominent neurological abnormalities, including locomotor defects and temperature-sensitive paralysis phenotype, suggesting defective neural transmission and excitability (10, 39, 144), the phenotypes analogous to neurological abnormalities associated with sialylation defects in mammalian nervous system. *Drosophila* sialylation genes show synergistic genetic interactions with *Para*, the gene encoding the *Drosophila* Na_V_ channel. Consistent with the effects of sialylation on vertebrate Na_V_ channels, DSiaT knockout alters the functional properties of Para and results in its posttranscriptional downregulation (39, 144), indicating that sialylation-mediated regulation of voltage-gated channels represents one of the most ancient and evolutionarily conserved functions of neural sialylation in animals. CSAS inactivation renders *Drosophila* highly sensitive to heat and oxidative stress, while transgenic CSAS upregulation increases stress tolerance beyond WT levels (144). This indicates that CSAS activity serves as a pathway bottleneck and suggests that CSAS plays a regulatory role in neural sialylation. Remarkably, the last two steps of the sialylation pathway mediated by CMP-sialic acid synthetase and sialyltransferase (Fig. 1) are separated in *Drosophila* between different cell types, as CSAS functions in glial cells, while DSiaT expression is restricted to neurons (39, 144). Such a bipartite arrangement of *Drosophila* sialylation provides a novel mechanism of glia-neuron coupling, allowing glial cells to directly regulate neuronal sialylation and modulate neural transmission (Fig. 4). It is tempting to speculate that this mechanism is potentially conserved in mammals, however, further studies are required to test this hypothesis. Unlike its mammalian counterparts that are localized to the nucleus, CSAS in *Drosophila* and other arthropods localizes to the secretory compartments, predominantly the Golgi and the endoplasmic reticulum (9, 10, 11, 145). Despite this difference in subcellular localization, the human ortholog of CSAS can fully rescue *Drosophila CSAS* mutants, highlighting the evolutionary conservation of CSAS function from flies to humans (145). However, the molecular targets of sialylation are not well-characterized in *Drosophila* and mechanisms of their functions remain to be elucidated.

## Sialylation in neurological disorders

**Figure 5:**
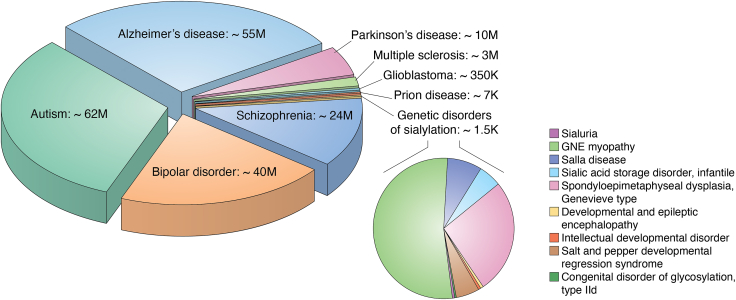
**Global prevalence of neurological disorders associated with abnormal sialylation or sialic acid–dependent pathological mechanisms.** Estimated numbers are based on information provided by World Health Organization, Alzheimer's Disease International, Parkinson's Foundation, National Multiple Sclerosis Society, and peer-reviewed publications (208), (https://www.who.int/, https://www.alzint.org/about/dementia-facts-figures/dementia-statistics/, https://www.parkinson.org/understanding-parkinsons/statistics, https://www.nationalmssociety.org/about-the-society/who-we-are/research-we-fund/ms-prevalence), (209).

Sialic acid–mediated molecular and cellular interactions are essential for bone development, skeletal growth, brain development, learning and memory formation, and immune responses (7, 31, 32, 146, 147). Dysregulation of sialylation can lead to aberrant cell–cell interactions, altered signaling processes, and changes in cell surface properties that can contribute to various conditions, including inflammation, infectious and cardiovascular diseases, and cancer (146). Abnormal sialylation is closely associated with various neurological and neurodegenerative disorders, as well as the progression of brain cancers, particularly glioblastoma. These conditions can be broadly classified into two main groups: (*i*) genetic disorders resulting from pathogenic variants of the genes directly involved in the sialylation pathway and (*ii*) diseases, often of unknown etiology, associated with mechanisms involving sialylation. This second group includes disorders characterized by abnormal sialylation in the absence of identified genetic mutations within the sialylation pathway (Table 1, Fig. 5). In our review, we will primarily focus on the second category of diseases. For a comprehensive discussion of genetic disorders directly linked to the sialylation pathway, we refer readers to the accompanying review on the genetics of sialylation (Huang *et al. J Biol Chem, in press*).

**Table 1:** Disorders of the nervous system associated with pathomechanisms involving sialylation

Disease or Abnormality	*Affected gene* & protein	Phenotypes & clinical features	Pathomechanisms & sia involvement
Sialuria MIM # 269921	*GNE* UDP-GlcNAc 2-epimerase/ManNAc kinase	Hepatosplenomegaly, coarse facial features, macrocephaly, mild intellectual impairment, and varying degrees of developmental delay	Dominant negative mutations causing failure of negative feedback inhibition by CMP-Sia. Associated with dramatic increase of free sialic acid levels (210)
GNE myopathy (Nanaka myopathy) MIM # 605820	*GNE*	Progressive adult-onset myopathy affecting the lower limbs and resulting in gait abnormalities or loss of ambulation. Occasional neurological involvement.	Loss of function missense mutations, reduced enzymatic activity. Hyposialylation and destabilization of muscle membrane glycoproteins, impaired cell interactions (34).
Salla disease Sialuria, adult form MIM# 604369	*SLC17A5* Sialic acid transporter, sialin (Solute carrier family 17 sodium phosphate cotransporter member 5)	Hypotonia, cerebellar ataxia, intellectual impairment, progressive psychomotor retardation of early onset, impaired speech, slowly progressing neurodegenerative conditions. Adult form of sialuria.	Loss of function mutations. Sialic acid lysosomal storage abnormality due to deficient transport of free sialic acid at the lysosomal membrane (211).
Infantile sialic acid storage disease, ISSD Sialuria, infantile form MIM# 269920	*SLC17A5*	Hypotonia, cerebellar ataxia, and mental deficiency; visceromegaly and coarse face features, progressive cerebellar atrophy, and dysmyelination revealed by MRI.	Loss of function mutations. Allelic to Salla disease, shared mechanisms. Abnormally enlarged lysosomes, accumulation of free sialic acid (212).
Spondyloepimetaphyseal dysplasia, Genevieve type MIM # 610442	*NANS* N-Acetylneuraminic acid phosphatase synthase	Infantile-onset severe developmental delay and skeletal dysplasia, short stature, severe mental deficiency, microcephaly, and ataxia.	Loss of function alleles, decreased level of NANS activity. Elevated level of N-acetyl-D-mannosamine in body fluids, but normal sialylation of plasma proteins (31)
Developmental and epileptic encephalopathy 15, DEE15, West syndrome MIM # 615006	*ST3GAL3* α2-3 Sialyl-transferase 3	Developmental and epileptic encephalopathy, seizures, hypotonia, primitive reflexes.	Loss of function mutations, reduced activity of ST3GAL3 that forms the sialyl Lewis-a (sLe^a^) epitope involved in a variety of cellular communication processes (32, 213).
Intellectual developmental disorder, autosomal recessive 12 (MRT12) MIM # 611090	*ST3GAL3*	Nonsyndromic impaired intellectual development, behavioral abnormalities, difficulty walking, loss of acquired skills.	Loss of function missense mutations affecting ST3GAL3 activity. Allelic to DEE15 (32).
Salt and pepper developmental regression syndrome MIM # 609056	*ST3GAL5* α2-3 Sialyl-transferase 5	Infantile onset of refractory and recurrent seizures, profoundly delayed psychomotor development and/or developmental regression, abnormal movements, and vision loss.	Loss of function mutations affecting the activity of ST3GAL5 responsible for the biosynthesis of GM3 gangliosides. Associated with the loss of GM3 and its derivatives, along with increased levels of lactosylceramide and its alternative derivatives (214).
Congenital disorder of glycosylation, type IIf MIM # 603585	*SLC35A1* CMP-sialic acid transporter	Psychomotor delay, seizures, dysmorphic features, hypotonia, intellectual disability, thrombocytopenia.	Loss of function mutations affecting the CMP-Sia transporter activity. Decreased sialylation of N- and O-linked glycans (33, 215, 216)
Congenital disorder of glycosylation, type IId MIM # 607091	*B4GALT1* β1-4-Galactosyl-transferase 1	Severe psychomotor and mental deficiency.	Loss of function mutations affecting B4GALT1 activity. Elevation of serum asialotransferrin, decreased galactosylated and sialylated structures (217, 218).
Neurodegenerative Diseases			
Spastic paraplegia-75 (SPG75) MIM # 616680	*SIGLEC-4/MAG* Myelin-associated glycoprotein (cell adhesion protein involved in myelin maintenance and glia–axon interaction)	Slowly progressing neurodegeneration, childhood-onset spastic paraplegia, and cognitive impairment. Atrophy of the corpus callosum and cerebellum, white matter abnormalities; Myelination defects in selected brain regions.	Uncharacterized missense and loss of function mutations inducing MAG degradation. Impaired myelination is the main proposed pathomechanism (201).
Multiple sclerosis	Unknown genetic causes	Progressive neurodegeneration. Vision, balance and coordination issues, muscle weakness.	Autoimmune-induced damage of myelin sheath within CNS. Abnormal sialylation of IgGs promoting neuroinflammation as a putative mechanism (177, 178, 219).
Parkinson’s disease	Unknown genetic causes	Progressive neurodegenerative disorder primarily affecting movement. Difficulties with motor skills, tremors, rigidity, bradykinesia, and postural instability.	Degeneration of nigrostriatal dopaminergic neurons, aggregation of misfolded α-synuclein, oxidative stress, neuroinflammation. Abnormal sialylation of peripheral IgGs indicating elevated antibody-dependent cell cytotoxicity (179). Potential ganglioside deficiency (reviewed in (220))
Alzheimer’s disease	*SIGLEC-3/CD33* *SIGLEC-11* *SIGLEC16 SIGLEC14 SIGLEC5 SIGLEC-2/CD22* Sialic acid–binding immunoglobulin-like lectins *APOE ε4 allele* Apolipoprotein E isoform 4	Progressive neurodegenerative disorder that primarily affects memory, thinking, and behavior.	Accumulation of Aβ, amyloid plaques. Hyperphosphorylation of tau, neurofibrillary tangles. Synaptic disfunction, neuroinflammation. GWAS-identified siglec variants associated with predisposition or protection. Aβ binding to clustered Sia potentially causes aggregation and amyloid plaques (reviewed in (161)). Sialylation-mediated effect on ApoE (173, 175, 176).
Psychiatric Disorders			
Autism spectrum disorders Bipolar disorders Schizophrenia	*ST8SIA2* α2-8-Sialyltrans-ferase 2 *SIGLEC-4/MAG* Myelin-associated glycoprotein	Mental anomalies, behavioral abnormalities, disorganized thinking, social-emotional anomalies.	Pathomechanism is unknown. *ST8SIA2:* Loss of function variants, reduced levels of polySia; uncharacterized polymorphisms; copy number variants (191, 192, 193, 194, 195, 196). *MAG:* polymorphisms (201, 202). Potential defects in MAG function.
Acute nervous system injury	Various or unknown etiology	Numbness, weakness, paralysis, vision problems, speech difficulties, severe headache, or loss of consciousness	Axonal and myelin fragmentation following injury. Expression of polySia promotes regrowth and repair (121, 205).
Prion diseases	*PRNP* Prion protein (PrPC)	Personality changes, psychiatric problems, myoclonus, insomnia, confusion, or memory loss, dementia	Misfolding of a normal or mutant prion protein into abnormal β-sheet–rich PrP-scrapie (PrP^Sc^) isoform, neurotoxic amyloid fibrils. Sialylation affects PrP^Sc^ infectivity and abnormal microglia activation (186, 221)
Glioblastoma, grade IV glioma	Multiple genetic lesions	Worsening headaches, nausea and vomiting, blurred or double vision, trouble speaking, altered sense of touch, and seizures.	Multiple genetic alterations, abnormal cell proliferation. Hypersialylation, increased expression of polySia-NCAM, ST6GAL1-mediated abnormal sialylation causing aberrant cell signaling and interactions (158, 222).

### Genetic disorders caused by mutations in the genes of the sialylation pathway

Pathogenic variants in genes implicated in the sialylation pathway result in rare conditions characterized by defects in different steps of the biosynthetic pathway of sialylation, recycling, and catabolism of sialic acids. These disorders commonly affect the nervous system but vary in clinical presentations, ranging from mild to severe forms. Known conditions involving neurological phenotypes include sialuria and GNE myopathy (MIM: 605820, 269921), free sialic acid storage disorders (MIM: 604369, 269920), spondyloepimetaphyseal dysplasia, Genevieve type (MIM: 610442), *ST3GAL3*-related neurodevelopmental disorder (MIM: 615006, 611090), *ST3GAL5*-related neurodevelopmental disorder (MIM: 609056), *SLC35A1*-related congenital disorder of glycosylation (MIM: 603585), and *CMAS* defect-associated intellectual disability (a single case reported so far requires further confirmation, no OMIM entry (35)). Some other congenital disorders of glycosylation can also involve defects in sialylated glycans and be associated with the phenotypes expected to result from abnormal sialylation, such as *B4GALT1* deficiency (MIM: 607091). These conditions typically present with developmental and intellectual delays, hypotonia, failure to thrive, facial dysmorphism, organomegaly, and seizures, underscoring the critical role of proper sialylation in maintaining normal organ functions, particularly in the nervous and muscular systems. Genetic testing and biochemical analysis of sialic acid levels in various tissues and fluids are important for the diagnosis. Currently, treatments are mainly supportive, mostly focusing on symptom management.

### Neurological diseases with pathomechanisms involving sialylation

Abnormal sialylation is a hallmark of several other neurological diseases that are not directly caused by pathogenic variants in the sialylation pathway genes. Most of these diseases currently have very limited therapeutic options (reviewed in (148, 149)).

#### Brain cancer

Aberrant sialylation, particularly hypersialylation, is a characteristic feature observed across multiple neoplasms, including gliomas and other malignant tumors, such as breast, lung, ovarian, and pancreatic cancers. Hypersialylation facilitates tumor progression by promoting immune evasion mechanisms, augmenting invasive and migratory capacities of cancer cells, and increasing cellular survival rates (*e.g.* reviewed in (150, 151)). Human glioma cells commonly have increased sialylation, which correlates negatively with disease prognosis. Changes in the expression of several sialylation pathway genes were found to affect glioma cells *in vitro* and in mouse models, although the mechanisms of these effects remain not well-understood (152, 153, 154). Glioblastoma, a grade IV glioma, and one of the most aggressive types of brain cancer, commonly exhibits increased expression of polySia-NCAM. This expression correlates with the upregulation of oligodendrocyte lineage transcription factor 2 (OLIG2), an essential factor for gliogenesis, suggesting a potential causative link to cancer progression (155). Silencing of Neu3 was found to potentiate invasiveness of glioblastoma cells associated with calpain activation, accumulation of GM3 gangliosides, and disassembly of focal adhesion (156). A key role of ST6GAL1-mediated α2-6 sialylation was demonstrated in promoting cell-renewal capacity of glioblastoma brain tumor-initiating cells in mouse models, suggesting tumorigenic effects of sialylation on platelet-derived growth factor receptor beta, activated leukocyte cell adhesion molecule (ALCAM), and Neuropilin (157). It is noteworthy that both the extent of sialylation and the turnover of sialylated glycans are significantly upregulated in glioblastoma. This turnover influences the connectivity within the glioblastoma cell network and may represent a promising target for therapeutic intervention (158).

#### Neurodegenerative conditions

##### Alzheimer's disease

AD is the most common cause of dementia and represents a significant healthcare burden. The AD pathogenesis involves the accumulation of extracellular deposition of insoluble beta-amyloid (Aβ) plaques and the intracellular aggregation of hyperphosphorylated tau protein in neurofibrillary tangles, accompanied by neuronal and synaptic degeneration (159). Although the relationship between sialylation and AD is not well understood, several lines of evidence suggest that abnormalities in sialylation may contribute to AD etiology. For instance, large-scale genome-wide association studies have identified variants in siglecs as predisposition factors for developing AD or as protective factors against it (*e.g.*, reviewed in (160, 161)). A genetic variant of CD33/Siglec-3 that results in truncation of the sialic acid–recognizing V-set domain was found to be protective against late-onset AD, while the full-length CD33M form was associated with an increased risk of developing AD (162, 163, 164). Similarly, Siglec-8 and its mouse counterpart Siglec-F were found to play important roles in regulating microglial activation during AD and in mouse AD models, respectively, potentially serving as a pathological mechanism that contributes to the onset and progression of AD and other types of dementia (165).

Further evidence linking sialylation to AD emerged from studies of human apolipoprotein E (ApoE), a lipid transport protein implicated in neurodegenerative disorders. The *APOE4* allele shows a strong genetic association with late-onset AD (166, 167), and its protein product was found to be a prominent disease marker across multiple neurodegenerative disorders, including AD, Parkinson's disease (PD), amyotrophic lateral sclerosis, and frontotemporal dementia (168). Conversely, the *APOE2* allele confers protective effects against late-onset AD (169). ApoE isoforms undergo modification with sialylated O-glycans (170, 171, 172), with ApoE2 exhibiting markedly greater sialylation levels than ApoE4 (173). Crucially, ApoE interacts with Aβ and contributes to pathogenic Aβ metabolism in AD (reviewed in (174)). Notably, sialylation of ApoE affects its binding to Aβ as well as ApoE-dependent Aβ fibrillation and microglia-mediated degradation in an isoform-specific manner (173, 175, 176), suggesting that sialylation regulates ApoE function under both healthy and pathological conditions.

##### Multiple sclerosis and Parkinson's disease

Multiple sclerosis (MS) is an inflammatory autoimmune demyelinating disease affecting the CNS and the most common cause of neurological disability in young adults in the North Temperate Zone. Although the etiology of MS remains poorly understood, studies have shown that abnormally low levels of sialylated intrathecal immunoglobulin G (IgG)s and increased monogalactosylated intrathecal IgG are the prominent traits associated with enhanced proinflammatory activity of IgGs in MS (177, 178).

A potential association between abnormal sialylation and PD, a complex progressive neurodegenerative disorder characterized by tremor, bradykinesia, and postural instability, has also been identified. PD progression was found to be associated with abnormal sialylation of transferrin and peripheral IgGs, indicating that increased antibody-dependent cell cytotoxicity may play a substantial role in the underlying pathomechanisms (179, 180).

An additional strong connection between neural sialylation and PD was revealed in studies on GM1 gangliosides. These sialylated glycosphingolipids were found to interact with α-synuclein and prevent its aggregation, thus protecting neurons from α-synuclein neurotoxicity (181). GM1 replacement therapy has been shown to slow PD-associated neurodegeneration in preclinical and some clinical studies, highlighting the important potential of this therapeutic strategy (*e.g.*, (182), reviewed in (183)). We refer readers to the accompanying review on gangliosides for a thorough discussion of their involvement in neurodegenerative conditions (Mlinac-Jerkovic *et al. J Biol Chem, in press*).

##### Prion diseases

Prion diseases or transmissible spongiform encephalopathies are a class of lethal, transmissible neurodegenerative disorders caused by misfolded, self-replicating pathogenic forms of the prion protein (PrP) referred to as PrP^Sc^. PrP^Sc^ can spread between cells and even organisms *via* recruiting host PrPs (PrP^C^) that help replicate the pathogenic misfolded forms (184). PrP^C^ is a glycoprotein modified with glycophosphatidylinositol anchor and normally carrying diverse glycoforms of sialylated N-linked glycans (185). The injection of desialylated PrP^Sc^ with exposed galactose residues resulted in prion clearance and inability to induce prion disease in animal models, indicating that sialylation is essential for PrP^Sc^ survival and infectivity within an organism (186, 187). There is an emerging evidence that sialylated N-linked glycans of PrP^Sc^ are key players in the pathogenesis of prion diseases at initial stages (reviewed in (188)), while abnormal microglia activation and neuronal engulfing may play important role at late stages that precede clinical onset (189).

#### Psychiatric disorders

Schizophrenia, bipolar disorder, and autism are serious psychiatric conditions characterized by shared genetics and molecular pathophysiology (190). Genome-wide association studies identified genetic variants in enzymes associated with sialic acid metabolism in these conditions. Dysregulation of polySia expression has been implicated in schizophrenia (134), while loss-of-function and several other alleles with single nucleotide and copy number variants of *ST8SIA2*, a polysialyltransferase gene, were found to be associated with schizophrenia (191, 192, 193, 194), bipolar disorder (191), and autism (195, 196). Consequently, individuals with schizophrenia frequently demonstrate reduced polySia levels (197, 198, 199), while mouse models deficient in ST8Sia2 display schizophrenia-like behaviors (200).

Psychiatric disorders can be mechanistically linked to neurodegeneration. For example, several loss of function mutations in *MAG*, which codes for Siglec-4/myelin-associated glycoprotein, are associated with autosomal recessive spastic paraplegia, a slowly progressive neurodegenerative disorder, while *MAG* polymorphisms have been linked to schizophrenia (201, 202). MAG is expressed early in development at the interface between myelinated axons and periaxonal myelin membrane and is required for normal myelination and axon development, and defects in these processes are thought to underlie the pathomechanisms of MAG-associated disorders (203, 204).

### Acute nervous system injury

An acute nervous system injury can manifest as a sudden onset of various clinical symptoms ranging from numbness and weakness to paralysis and loss of consciousness. Sialylation plays a crucial role in acute nervous system injuries, influencing both injury mechanisms and potential recovery processes. Local upregulation of polySia can support regrowth, neuronal migration, and enhance neural connectivity, indicating that increased polySia expression creates an environment that promotes architectural remodeling and repair of the nervous system (see also discussion of polySia functions above) (121, 205, 206).

### Potential therapeutic implications

Considering the essential roles of sialylation in healthy and diseased states of the nervous system, exploring new approaches that allow us to modulate neural sialylation along with associated signaling pathways constitutes a promising avenue for therapeutic intervention in neurological disorders. For example, targeting sialylation has been suggested as a potential therapeutic strategy for glioblastoma, a highly aggressive brain tumor with no effective treatment available to date (158). Notably, the use of antibodies that inhibit P-selectin, a sialic acid-binding lectin involved in cell adhesion, has progressed to clinical trials for the treatment of brain tumor metastases (https://clinicaltrials.gov/study/NCT05909618). Polymers of sialic acids have emerged as a novel therapeutic strategy for neurodegenerative conditions, and they have been successfully applied in humanized transgenic mice expressing Siglec-11 in mononuclear phagocytes, which ameliorated laser-induced damage of the retina in the age-related macular degeneration model (116). The therapeutic potential of targeting siglecs has been demonstrated by a number of studies (160). A notable example of applying antibodies to block CD22 function and reprogram microglia for a homeostatic state was demonstrated in experiments that restored cognitive function in aged mice (109). Yet another novel approach utilizing sialic acid–functionalized dendrimeric polymers has been effectively used to mimic cell surface sialylated glycoconjugates. This promising methodology demonstrated the ability of these sialic-acid-conjugated dendrimers to compete with Aβ binding to gangliosides and to reduce associated cytotoxic effects in cultured neuroblastoma cells (207). However, despite advancements in sialylation research and numerous successful proof-of-concept experiments illustrating the therapeutic potential of sialylation modulation, the detailed mechanistic connections between aberrant sialylation and neurological diseases remain not fully understood. Consequently, the current strategies targeting sialylation largely remain at the preclinical stage. An in-depth discussion of sialylation-related therapeutic approaches in cancer can be found in the accompanied review on this topic.

## Conclusions

Recent studies in various model systems and analyses of disease etiologies have uncovered new functions of sialylation in the nervous system. These findings indicate that known functions of neural sialylation represent just “a tip of the iceberg”, and ongoing studies are expected to reveal additional important mechanisms mediated by sialylation. Notably, these novel data have identified several unexpected characteristics of sialylation, including a dynamic interplay between sialylation and desialylation in neural transmission, as well as potential important roles of sialylated glycoRNA in neural regulation. New results have also elucidated mechanisms of several important known roles of sialylated glycans that were previously poorly understood, including the intertwined relationship between mechanisms of immune regulation and neural physiology. Intriguing questions about novel conserved mechanisms of sialylation-mediated interactions between glia and neurons have been posed by studies in the *Drosophila* model system. Many of these advances have been achieved due to the development of novel tools and approaches that allowed researchers to look deeper into mechanisms at several levels, ranging from organism behaviors and genetics to cellular and molecular details of Sia-mediated functions. These new mechanistic insights are expected to have important implications for clinical research and the development of novel therapies for neurological disorders associated with abnormal sialylation. Such expectations have been supported by proof-of-principle studies in model systems and advancement of some preclinical research to clinical trials. For example, mouse models demonstrated that the application of soluble polySia could protect from neurodegeneration; antibody-mediated inhibition of siglec functions prevented aging-associated cognitive decline in mice, while inhibition of Sia-selectin binding is currently at initial phases of clinical trials for brain metastases. Considering the wide spectrum of Sia functions in the nervous system, including involvement in neural development and physiology, affecting neural network formation, learning and memory, stress responses, aging and neurodegenerative conditions, it is anticipated that biomedical research in these areas will unveil new Sia-related therapeutic strategies for neurological conditions. At the same time, taking into account the overwhelming complexity of the mammalian brain and intricate glycosylation pathways in mammalian organisms, the studies in various simplified model systems are expected to continue playing the leading role, helping unravel conserved functions and mechanisms of neural sialylation in human health and disease.

## Conflicts of interest

The authors declare that they have no conflicts of interest with the contents of this article.
